# Effects of Antibiotic Residues on Fecal Microbiota Composition and Antimicrobial Resistance Gene Profiles in Cattle from Northwestern China

**DOI:** 10.3390/microorganisms13071658

**Published:** 2025-07-14

**Authors:** Wei He, Xiaoming Wang, Yuying Cao, Cong Liu, Zihui Qin, Yang Zuo, Yiming Li, Fang Tang, Jianjun Dai, Shaolin Wang, Feng Xue

**Affiliations:** 1 MOE Joint International Research Laboratory of Animal Health and Food Safety, College of Veterinary Medicine, Nanjing Agricultural University, Nanjing 210095, China; 2 Ningxia Hui Autonomous Region Food Testing and Research Institute, Yinchuan 750002, China; 3 National Key Laboratory of Veterinary Public Health and Safety, College of Veterinary Medicine, China Agricultural University, Beijing 100193, China; 4 Jiangsu Academy of Agricultural Science, Nanjing 210014, China; 5 School of Engineering, China Pharmaceutical University, Nanjing 210009, China; 6 Key Laboratory of Animal Antimicrobial Resistance Surveillance, Ministry of Agriculture and Rural Affairs, Beijing 100081, China; 7 Technology Innovation Center for Food Safety Surveillance and Detection (Hainan), Sanya Institute of China Agricultural University, Sanya 572025, China

**Keywords:** grazing cattle, antibiotic residues, microbial communities, antibiotic resistance genes

## Abstract

Grazing is a free-range farming model commonly practiced in low-external-input agricultural systems. The widespread use of veterinary antibiotics in livestock farming has led to significant environmental accumulation of antibiotic residues and antibiotic resistance genes (ARGs), posing global health risks. This study investigated the antibiotic residues, bacterial community, ARG profiles, and mobile genetic elements (MGEs) in cattle feces from three provinces in western China (Ningxia, Xinjiang, and Inner Mongolia) under grazing modes. The HPLC-MS detection showed that the concentration of tetracycline antibiotics was the highest in all three provinces. Correlation analysis revealed a significant negative correlation between antibiotic residues and the diversity and population abundance of intestinal microbiota. However, the abundance of ARGs was directly proportional to antibiotic residues. Then, the Sankey analysis revealed that the ARGs in the cattle fecal samples were concentrated in 15 human pathogenic bacteria (HPB) species, with 9 of these species harboring multiple drug resistance genes. Metagenomic sequencing revealed that carbapenemase-resistant genes (*bla*_KPC_ and *bla*_VIM_) were also present in considerable abundance, accounting for about 10% of the total ARGs detected in three provinces. Notably, *Klebsiella pneumoniae* strains carrying *bla*_CTX-M-55_ were detected, which had a possibility of IncFII plasmids harboring transposons and IS19, indicating the risk of horizontal transfer of ARGs. This study significantly advances the understanding of the impact of antibiotic residues on the fecal microbiota composition and ARG profiles in grazing cattle from northwestern China. Furthermore, it provides critical insights for the development of rational antibiotic usage strategies and comprehensive public health risk assessments.

## 1. Introduction

Grazing-based livestock production is the dominant form of animal husbandry in northwestern China, covering approximately 40% of the country’s land area [1]. This region plays a crucial role in beef cattle production, with an annual output of 21.4 million cattle, primarily in Xinjiang, Ningxia, and Inner Mongolia [2]. Compared to intensive farming, grazing systems are generally considered to have lower environmental health risks [3]. However, the widespread use of antibiotics in livestock production raises concerns regarding antibiotic residues in grazing environments and their potential impact on gut microbiota, antibiotic resistance genes (ARGs), and mobile genetic elements (MGEs).

Antibiotics are extensively used in animal husbandry to promote growth and prevent infectious diseases, yet only about 15% of administered antibiotics are metabolized and assimilated by animals [4]. The majority are excreted through feces, introducing antibiotic residues into the broader ecosystem via fertilization, sewage irrigation, and surface runoff [5]. The levels of antibiotic residues vary depending on species and husbandry practices [6]. Incomplete antibiotic absorption and subsequent environmental excretion contribute to the emergence of antibiotic-resistant bacteria in agricultural settings [7,8]. While the impact of antibiotic residues on ARG dissemination in intensive farming has been extensively studied, systematic investigations in grazing systems remain limited [9].

The overuse of antibiotics not only disrupts the gut microbiota of livestock by reducing bacterial diversity and altering community structure but also promotes the proliferation of antibiotic-resistant bacteria [10]. These disturbances affect the intestinal health of both animals and humans and elevate antibiotic resistance levels in pastures and surrounding environments [11]. Bacteria acquire resistance primarily by encoding ARGs, which complicates the treatment of bacterial infections in both humans and animals [12,13,14]. Increasing evidence highlights the presence of clinically relevant ARGs, such as *mcr*, *bla*_KPC_, *bla*_NDM_, and *bla*_CTX-M_, in livestock environments [15,16]. Genetic analyses have revealed a high degree of similarity between ARG profiles of human pathogens and those of livestock-origin bacteria, suggesting potential cross-species ARG transmission [17]. Consequently, livestock farming—including cattle, swine, and poultry production—is recognized as a major source of ARG contamination [18]. Despite the growing interest in ARG dissemination in grazing systems [5], comprehensive studies on ARG transmission within grazing cattle and their environment remain scarce.

Grazing systems influence the prevalence of antibiotic-resistant microorganisms through fecal deposition. This deposition acts as a significant source of antimicrobial resistance (AMR)-related elements in the air, including antibiotic resistance genes (ARGs), human pathogenic bacteria (HPBs), and mobile genetic elements (MGEs). These elements can affect soil microbiota. Furthermore, fecal deposition contributes to the dispersal of resistant bacteria via bioaerosols and plant–soil interactions [7,19,20,21]. However, the ARG in grazing environments remains poorly understood. The microbial taxa driving ARG distribution and the extent to which grazing conditions influence ARG transmission are still unclear, particularly across diverse microhabitats [20].

To address these gaps, this study investigates fecal antibiotic residues and ARG composition in grazing cattle from three northwestern Chinese provinces: Xinjiang, Ningxia, and Inner Mongolia. Given that Xinjiang and Inner Mongolia rank among the top four grazing regions in China, and Ningxia also has substantial grazing resources [2], these regions serve as ideal locations for assessing antibiotic resistance under extensive livestock management. Using metagenomic sequencing, we characterize the intestinal microbiota and ARGs in grazing cattle and explore the association between ARGs and MGEs. Additionally, we analyze the co-occurrence between key ARGs and marker genes of common pathogenic bacteria (e.g., *Escherichia coli* and *Salmonella* spp.) in the metagenomes to investigate potential associations and infer mechanisms of resistance dissemination among the gut microbiota. This study provides a comprehensive analysis of antibiotic residues, gut microbiota, and ARGs in grazing cattle from northwestern China. By elucidating the relationships between MGEs and ARGs, our findings contribute to a deeper understanding of antibiotic resistance transmission in grazing systems, offering critical data for developing sustainable livestock management strategies.

## 2. Materials and Methods

### 2.1. Sample Collection

**Figure 1:**
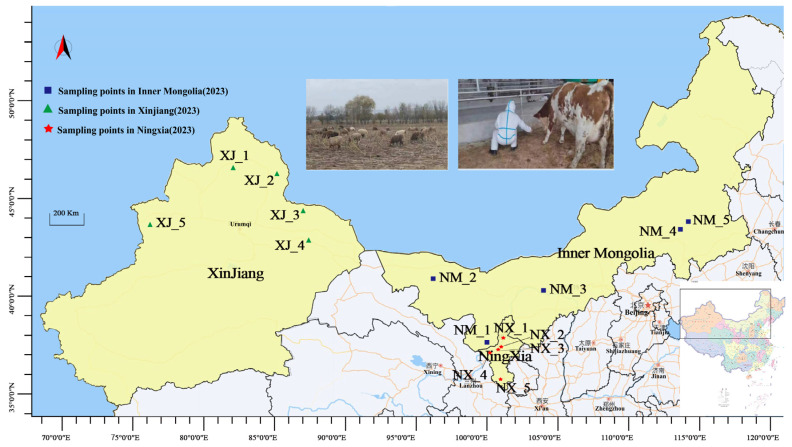
Map of geographical distribution of sample collection.

From August to October 2023, fresh cattle manure samples (200 g each) were aseptically collected directly from the floor using sterile gloves and disposable collection sleeves at 15 ranches across Xinjiang (XJ), Ningxia (NX), and Inner Mongolia (NM). The ranches were selected using simple random sampling (Figure 1). Cows were systematically selected by farm staff based on clinical health assessments (normal temperature, appetite, mobility, and absence of visible disease symptoms). From each ranch, 10 cows were randomly chosen from morning milking herds, ensuring representation across different pens and age groups. Manure samples were immediately placed in sterile 50 mL tubes, flash-frozen in liquid nitrogen within 15 min, transported on dry ice, and stored at −80 °C. To minimize environmental contamination, freshly dropped stool samples were collected. For homogeneity, equal aliquots from all 10 samples per ranch were combined into a single composite sample (labeled NX_1–NX_5, NM_1–NM_5, and XJ_1–XJ_5).

Furthermore, meteorological data from 15 ranches in 3 provinces were also collected in our research (Figure S1).

### 2.2. Analysis of Antibiotic Residues

The QuEChER method [22] was used to extract antibiotics from fecal samples. In brief, 2 mL of EDTA was added to the feces and vortexed for 1 min, then 8 mL of acetonitrile was added, 5 g of NaCl was added, and vortexed for 1 min. The sample was centrifuged at 9000 rpm at 4 °C for 5 min. Then, 4 mL of the supernatant was added to the QuEChER salt pack (Agilent Technologies, Beijing, China), vortexed for 1 min, and centrifuged at 9000 rpm at 4 °C for 5 min. The supernatant was nitrogen dried and reconstituted to 1 mL (reconstitution solution: 10% acetonitrile–water). The final solution was filtered into a glass sample bottle through a 0.2 μm WWPTFE membrane syringe filter (Waters, Shanghai, China) and analyzed by HPLC-MS.

The external standard method was used for quantification during the determination. The analysis was performed on an Agilent 6460C triple quadrupole mass spectrometer (Agilent Technologies, Beijing, China) coupled with an HPLC system for quality control and quantitative analysis of antibiotic residues. The corresponding standard curves were established with 8 standard solutions with concentrations of 0.1, 1.0, 5.0, 10, 20, 50, 80, and 100 ng/mL. We investigated the matrix effect (ME) of each target component in the fecal matrix. We prepared standard curves using fecal blank matrix solution and pure solvent solution, respectively, and calculated the ME value according to Table S1. The data were deemed credible, with a relative determination (R^2^) value greater than 0.999. A recovery rate experimental group was established during the measurement of the samples, and one sample was selected for repeated determination (*n* = 6). The results showed that the spiked recovery rates of all antibiotics in feces ranged from 40% to 115%, and the relative standard deviation (RSD) was <20% (Table S1). There was no significant difference in the recovery rates of fecal samples from the three regions. The sensitivity parameters, including LOD and LOQ, for all target antibiotics are summarized in Table S2. Briefly, LODs and LOQs were established at analyte concentrations yielding signal-to-noise ratios of approximately 3 and 10, respectively. A program blank was set during the experiment, and the data in the paper were all the results of deducting the program blank.

### 2.3. Metagenomic Sequencing

Total DNA from feces samples was extracted using the Magen Fecal Genomic DNA Extraction Kit (HiPure Soil DNA Mini Kit) (Guangzhou Magen Biotechnology, Guangzhou, China), and all extraction procedures were performed according to the instructions. DNA extracts were subjected to 1% agarose gel electrophoresis to identify the integrity of DNA. Qubit 3.0 (Thermo Fisher Scientific, Waltham, MA, USA) and NanoDrop1 (Thermo Fisher Scientific, Waltham, MA, USA) were used to determine the concentration and purity of DNA, and the extracted DNA was stored at −20 °C for subsequent studies.

The quality of the metagenomic library was assessed using the Qubit 4.0 Fluorometer (Life Technologies, Grand Island, NY, USA) and the Qsep400 High-Throughput Nucleic Acid and Protein Analysis System (BiOptic, Jiangsu, China), and the library was sequenced using the DNBSEQ-T7 platform of BGI Genomics Co., Ltd., Shenzhen, China (150 bp paired-end reads).

### 2.4. Library Construction and Sequencing

Shotgun metagenomic libraries were constructed using the DNBSEQ-T7-compatible protocols [23]. Briefly, qualified DNA was randomly fragmented, end-repaired, and ligated with platform-specific adapters. Library quality was assessed using the Qubit 4.0 Fluorometer (Life Technologies, Grand Island, NY, USA) and Qsep400 System (Bioptic, Jiangsu, China). Sequencing was performed on the DNBSEQ-T7 platform (BGI Genomics, Shenzhen, China) with 150 bp paired-end reads, leveraging its DNA Nanoball (DNB) technology (BGI Genomics, Shenzhen, China). High-throughput shotgun metagenomic sequencing provides a comprehensive approach for characterizing microbial community structure and functional potential.

### 2.5. Metagenomic Data Processing

Quality control used FASTP (v0.21.0) to trim sequencing adapters and filter low-quality reads [24]. MEGAHIT V1.1.2 was used to assemble the clean data after quality control of metagenomic data separately by sample [25], filter out fragments less than 300 bp in length in the assembly results, and then perform statistical analysis and subsequent gene prediction. Metagenomic data analysis relied on UHGG V2.0 and NCBI NT (20240112) databases, used Kraken2 to classify and annotate each contig, and then constructed species abundance based on Bracken 2.6.1.

Binning analysis was employed to identify and classify ARGs from metagenomic data. Contigs with a length of ≥1000 base pairs were selected as the final assembly results, ensuring sufficient sequence information for accurate binning. These contigs were subsequently subjected to genome binning to derive metagenomic assembly genomes (MAGs). The binning process utilized MetaBAT (version 2.12.1) and MaxBin2 (version 2.2.5), both tools designed to group contigs into coherent genomic bins based on coverage and tetranucleotide frequency patterns [26].

To assess the quality of the generated MAGs, CheckM (version 1.0.12) was applied to estimate the integrity, contamination, and strain heterogeneity of each bin. Integrity scores reflect the completeness of the genome bins, while contamination levels indicate the presence of foreign sequences. Strain heterogeneity evaluates the potential diversity within the bin. Bins with an integrity score exceeding 75% and contamination levels below 5% were retained for further analysis, ensuring high-quality MAGs that could reliably represent microbial genomes. This stringent filtering criterion aimed to minimize false positives and enhance the accuracy of downstream analyses. Using Pearson and Mantel tests, we inspected ARG and MGE spectra of the relationship, and visualized through the online web: https://www.chiplot.online/.

The relationship between microorganisms, ARGs, drug classes, and resistance mechanisms was quantified using Pearson’s correlation coefficients. To account for the increased risk of Type I errors due to multiple comparisons, we applied the Benjamini–Hochberg false discovery rate (FDR) correction [27,28]. Briefly, all calculated *p*-values from pairwise correlations were ranked in ascending order, and the significance threshold for each *p*-value was adjusted according to the formula:

$$p_{\begin{array}{c} \mathrm{adj} \end{array}}^{\left(i\right)}=p^{\left(i\right)}\times \frac{m}{\begin{array}{c} i \end{array}}$$

where *m* is the total number of comparisons and *i* is the rank of the *p*-value. Correlations with *p* < 0.05 were considered statistically significant. This approach controls the expected proportion of false discoveries to ≤5% while maintaining statistical power in large-scale comparisons.

### 2.6. Statistical Analysis

Diversity analysis results were visualized using the ggplot2 R package (version 3.6.0) [29]. Linear discriminant analysis (LDA) effect size (LEfSe) in ggplot2 was applied to the normalized ARG count data (relative abundance per sample) to identify signature ARGs, with significance thresholds of *p* < 0.05 and LDA score > 2. To investigate co-occurrence patterns among ARGs, pairwise Spearman’s rank correlations were calculated on the ARG relative abundance matrix using the correst function from the “psych” package (version 4.2.3). Correlation strength was assessed by the Spearman’s ρ coefficient, and robustness was defined by two criteria: (1) statistical significance (*p* < 0.05 after false discovery rate correction), and (2) an effect size threshold of R^2^ > 0.05 (ρ^2^ > 0.05), where R^2^ represents the squared Spearman correlation coefficient. Only robust correlations meeting both criteria were used to construct the co-relative network [30].

R^2^ > 0.05 indicates that >5% of the variance in one ARG’s abundance is explained by another, ensuring biologically meaningful associations beyond statistical significance.

A correlation matrix was calculated by calculating all possible pairwise Spearman’s rank correlations between the ARGs and MGEs/HPBs to visualize the correlations in the network interface. A correlation between two items was considered statistically robust if the Spearman’s correlation coefficient (∣ρ∣) was > 0.7 and *p* < 0.01 [31].

Source tracking analysis was conducted in R (version 3.6.3) [32] to quantify the contributions of diverse environmental sources to microbial communities and antibiotic resistance genes (ARGs) in cattle fecal samples from the western pastoral region (designated as sink samples), while microbial communities from diverse sampling points served as source samples. The analysis employed a Bayesian algorithm (adapted from variational Bayesian frameworks) for source separation to resolve microbial community composition, ARG profiles, and their respective source–sink contributions. Additionally, structural distribution patterns of microorganisms and ARGs were analyzed using principal component analysis (PCA) to identify co-occurrence networks and predict horizontal gene transfer mechanisms. To trace mobile genetic elements (MGEs) facilitating ARG dissemination, the analysis integrated (i) MGE–host linkage via covariance matrices and (ii) source-specific MGE enrichment scores. This multi-layered approach addressed three aims: (1) quantifying source–sink contamination ratios, (2) resolving ARG dissemination pathways, and (3) identifying high-risk MGE vectors for targeted intervention.

## 3. Results

### 3.1. Antibiotic Residues in Cattle Feces

**Figure 2:**
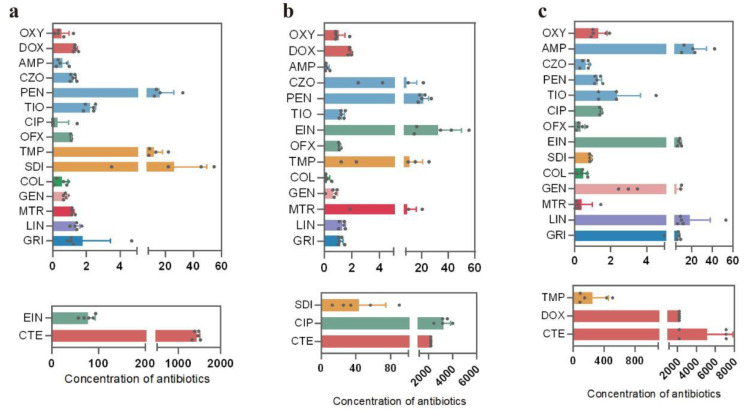
Detection values of antibiotic residues in cattle feces samples. (**a**) Antibiotic residue contents in the Xinjiang region, (**b**) antibiotic residue contents in the Inner Mongolia region, and (**c**) antibiotic residue contents in the Ningxia region. *n* = 5. Abbreviations: oxytetracycline (OXY), chlortetracycline (CTE), doxycycline (DOX), ampicillin (AMP), cefazolin (CZO), penicillin G (PEN), cefuroxime (TIO), ciprofloxacin (CIP), ofloxacin (OFX), enrofloxacin (EIN), trimethoprim (TMP), sulfadiazine (SDI), colistin (COL), gentamicin (GEN), metronidazole (MTR), lincomycin (LIN), and griseofulvin (GRI).

A total of 18 antibiotics belonging to 9 major categories (tetracyclines, sulfonamides, aminoglycosides, quinolones, β-lactams, peptides, antifungals, nitroimidazoles, and macrolides) were detected in cattle fecal samples (Figure 2). The concentration of tetracycline residues was the highest in all fecal samples. HPLC-MS showed that the average residues of chlortetracycline in Xinjiang, Inner Mongolia, and Ningxia were 1437.9 μg/kg (Figure 2a), 2176.95 μg/kg (Figure 2b), and 5158.4 μg/kg (Figure 2c), respectively. Doxycycline had the highest residual concentration (2179.1 μg/kg) in Ningxia samples (Figure 2c). Compared with the other two regions, Ningxia was the region with the highest concentration of tetracycline antibiotics.

Besides tetracyclines, enrofloxacin had the highest residual concentration (77.7 μg/kg) in Xinjiang samples, while ciprofloxacin had the highest residual concentration (3265.1 μg/kg) in Inner Mongolia samples. Compared with Ningxia, the samples from Xinjiang and Inner Mongolia had higher quinolone antibiotic content (Figure 2). Four types of β-lactam drugs were detected in samples from all three regions, namely, ampicillin, cefazolin, penicillin G, and cefuroxime. Among them, penicillin G exhibited the highest average concentrations in Xinjiang and Inner Mongolia, which were 16.2 μg/kg and 21.2 μg/kg, respectively. Samples from Ningxia showed that ampicillin had the highest concentration, reaching 22.0 μg/kg. In addition, sulfonamide residues were present in relatively low amounts in the samples from Xinjiang. However, sulfadiazine and trimethoprim were detected at considerably higher concentrations in the samples collected from Ningxia and Inner Mongolia. Specifically, the average concentration of sulfadiazine in the Inner Mongolia samples was measured at 43.8 μg/kg (Figure 2b), while the average concentration of trimethoprim in the Ningxia samples reached 256.7 μg/kg (Figure 2c). Among them, lincomycin had the highest residue level in Ningxia, reaching 18.2 µg/kg (Figure 2c). In general, the highest total concentration of antibiotics in cattle feces was in Ningxia, followed by Inner Mongolia and Xinjiang, which may be due to the correlation between antibiotic use and environmental factors in different regions.

### 3.2. Characteristics of the Intestinal Microbiota of Grazing Cattle in Northwestern China

**Figure 3:**
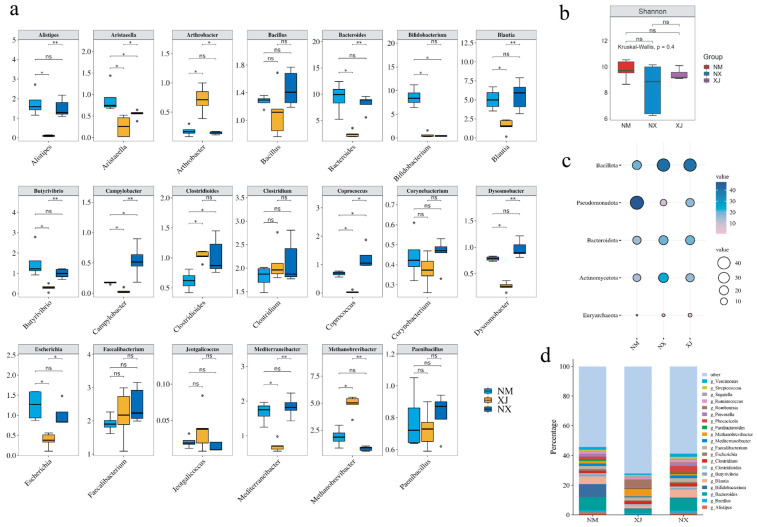
Analysis of cattle intestinal microbiota composition in three northwestern pastoral provinces. (**a**) Analysis of the inter-group differences at the genus level and the proportions of relative abundance at phylum levels among different samples from pastoral areas of Ningxia (NX), Inner Mongolia (NM), and Xinjiang (XJ). ns: *p* > 0.05, *: *p* < 0.05, and **: *p* < 0.01. (**b**) Alpha diversity analysis at the species level. (**c**) Phylum levels among different pastoral areas. In this diagram, the bubbles’ color from pink to blue represents the relative abundance of the different phyla from low to high, and the size of the circles also represents the abundance, the larger the higher. (**d**) Genus levels with a relative abundance greater than 1% among different pastoral areas.

MEGAHIT was harnessed to dissect metagenomic sequencing data to clarify the composition of the cattle intestinal microbiota in grazing regions. The results indicated that phyla *Bacillota*, *Pseudomonadota*, *Bacteroidota*, and *Euryarchaeota* were dominant in cattle intestinal microbiota, collectively comprising over 90% of the total bacterial community (Figure 3d). Ningxia (NX) samples exhibited higher abundances of *Bacteroidota* and *Bacillota* but lower abundance of *Pseudomonadota* than those from Inner Mongolia (NM) and Xinjiang (XJ) samples (Figure 3c). It is hypothesized that the abundances of *Bacteroidota* and *Bacillota* are positively correlated with antibiotic residues, while *Pseudomonadota* abundance is negatively correlated with antibiotic residues. Combining to the total antibiotic residues of three provinces (Figure 2), it seemed that the abundances of *Bacteroidota* and *Bacillota* were positively correlated with antibiotic residues, while *Pseudomonadota* abundance was negatively correlated with antibiotic residues.

Further investigation at the genus level showed that Xinjiang had a lower abundance than the other two provinces. Key genera contributing to these differences included *Romdoutsia*, *Bacteroides*, *Bifidobacterium*, *Phocaeicola*, and *Blautia* (Figure 3d).

Enrichment analysis identified 20 differentially abundant genera among 3 grazing areas. The abundance of opportunistic pathogen genera, such as *Bacillus*, *Clostridioides*, *Escherichia*, and *Streptococcus*, in the Xinjiang samples was significantly lower than those in Ningxia and Inner Mongolia samples (Figure 3a). All of these pathogen genera can be classified into the phyla *Bacillota* and *Bacteroidota*. Based on the previous antibiotic residue data in cattle feces samples, a positive correlation was observed between elevated antibiotic residues and higher abundances of opportunistic pathogenic genera in corresponding samples; conversely, non-pathogenic genera exhibited negative correlations with antibiotic residue levels (Figure 2 and Figure 3).

### 3.3. The Diversity of Intestinal Microbiota Is Strongly Associated with Antibiotic Use

**Figure 4:**
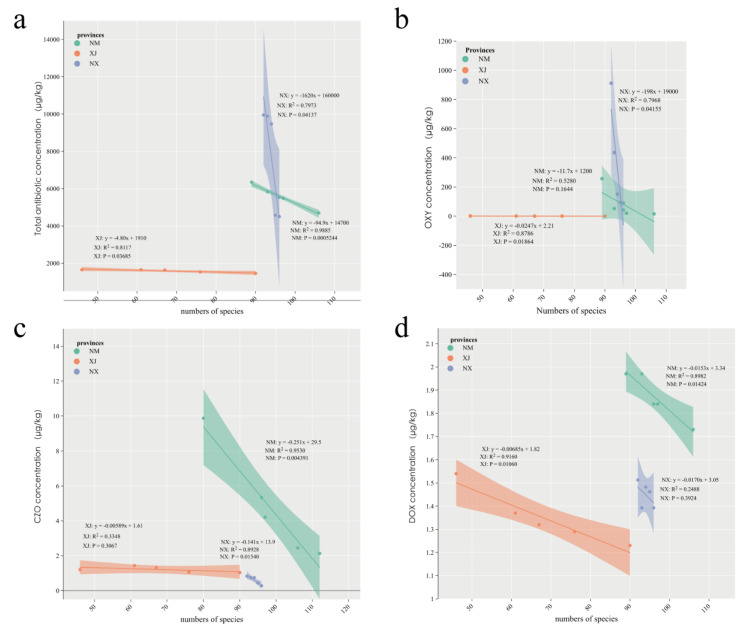
Correlation analysis between cattle intestinal microbial composition and antibiotic residues in three provinces. (**a**) Linear correlation analysis between the number of intestinal microbiota species and antibiotic residue concentrations in NX, NM, and XJ provinces. (**b**–**d**) Linear correlation analysis between intestinal microbiota species and oxytetracycline (**b**), cefazolin (**c**), and doxycycline (**d**) in three grazing provinces.

To further investigate the relationship between antibiotic residues and gut microbiota, we analyzed the correlation between bacterial composition and total antibiotic concentration in cattle feces using linear correlation analysis. The results revealed a negative correlation between microbiota abundance and total antibiotic concentration across all three provinces (Figure 4a). Specifically, in Xinjiang, the correlation coefficient, R^2^, was 0.8117 (*p* = 0.03685), in Inner Mongolia, R^2^ was 0.9885 (*p* = 0.0005), and in Ningxia, R^2^ was 0.7973 (*p* = 0.04137; Figure 4a). Notably, total bacterial species in all three provinces was negatively correlated with oxytetracycline (OXY) and doxycycline (DOX), both belonging to the tetracycline class, as well as cefazolin (CZO), a β-lactam antibiotic (Figure 4b–d). In Xinjiang, annotated total species exhibited a significant negative correlation with OXY and DOX (*p* = 0.01864 and *p* = 0.01060, respectively; Figure 4b,d). Additionally, a significant negative correlation was observed between total microbiota species in Inner Mongolia and CZO concentration (*p* = 0.0154; Figure 4c).

Overall, this study demonstrates that antibiotic residues significantly influenced the composition of the intestinal microbiota, highlighting the impact of antibiotic use on the microbial diversity of grazing cattle.

### 3.4. Relative Abundance of ARGs, MEGs, and HBPs

**Figure 5:**
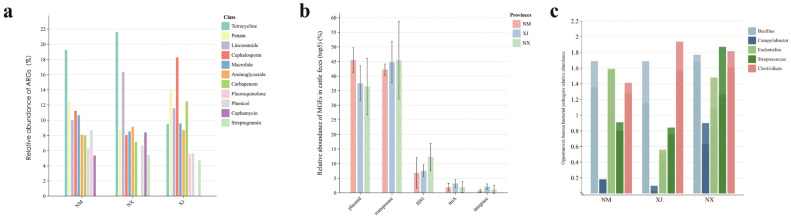
Relative abundance of ARGs, MGEs, and HPBs in cattle feces. (**a**) Analysis of the average distribution of ARGs, (**b**) analysis of distribution of MGEs, and (**c**) analysis of the distribution of the top 5 genera containing HPBs.

To further explore the transmission risks of ARGs, we proceeded to analyze the ARGs, mobile genetic elements (MGEs), and human pathogenic bacteria (HPBs) in cattle gut microbiota using metagenomic sequencing. A total of 43 ARG categories (comprising 705 individual ARGs), 3 major MGE types (integrons, transposons, and plasmids), and 5 HBPs (*Staphylococcus* spp., *Escherichia coli*, *Clostridium* spp., *Klebsiella pneumoniae*, and *Campylobacter* spp.) were annotated (Figure 5). The genera containing important HBPs (*Staphylococcus* spp., *Escherichia* spp., *Clostridium* spp., *Klebsiella* spp., and *Campylobacter* spp.) were annotated (Figure 5, top 5 listed).

Among the three pastoral regions, samples from Ningxia (NX) exhibited the highest abundance of both ARGs and HPBs (Figure 5a,c), followed by Inner Mongolia (NM) and Xinjiang (XJ). Combined with previous antibiotic residue data (Figure 2), these findings suggest a positive correlation between the total antibiotic concentration and the abundance of ARGs/HPBs in cattle fecal samples.

Notably, the high detection frequency of MGEs in northwestern pastoral areas (Figure 5b) indicates their widespread presence in grazing environments. Plasmids and transposons were predominant in cattle samples, whereas Xinjiang feces exhibited higher relative abundances of integrase and the transposase *tniA* compared to those in Ningxia and Inner Mongolia (Figure 5).

**Figure 6:**
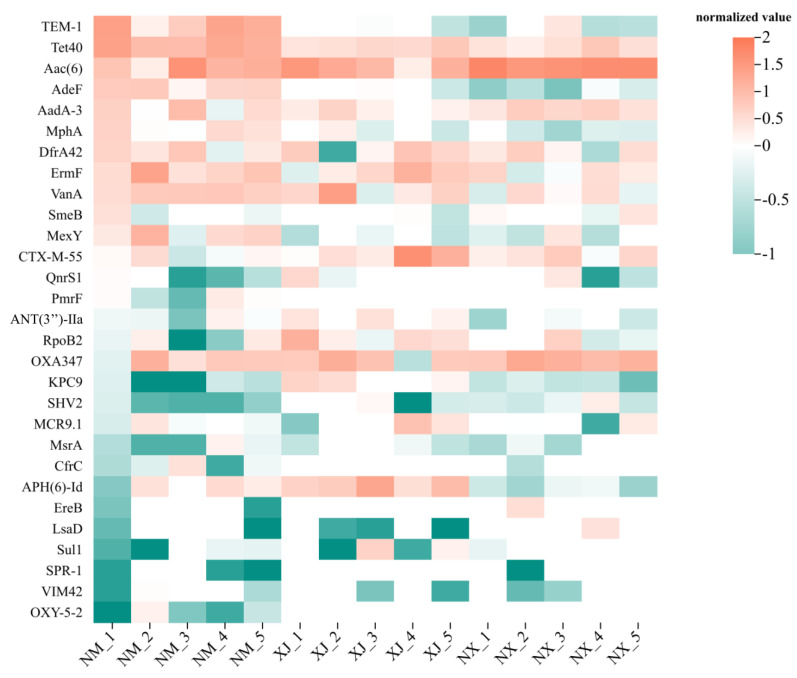
Clustering heat map of ARGs in grazing cattle feces samples. Distribution of MGEs in cattle feces samples from three regions.

To elucidate the relationship between antibiotic residues and ARGs and characterize the richness and diversity of ARG distributions in cattle fecal samples from different pastoral areas, a cluster analysis was performed on the top 30 ARGs from cattle fecal samples (Figure 6). Overall, the fecal samples from the Inner Mongolia (NM) pastoral area exhibited the highest abundance of ARGs, while those from the Xinjiang (XJ) pastoral area had the lowest. It was found that the ARGs for β-lactams were highly abundant in all three major pastoral areas. Further, *cfrc*, *msrA*, and *ereB* were the predominant resistance genes for MLSB-type antibiotics. Regarding *bla* genes encoding VIM45, CTX-M-55, SHV2, and OXA347 were the main ARGs for β-lactams. For aminoglycosides, *aad-3*, *ant(3″)-IIa*, and *aac(6′)* were the principal ARGs (Figure 6).

**Figure 7:**
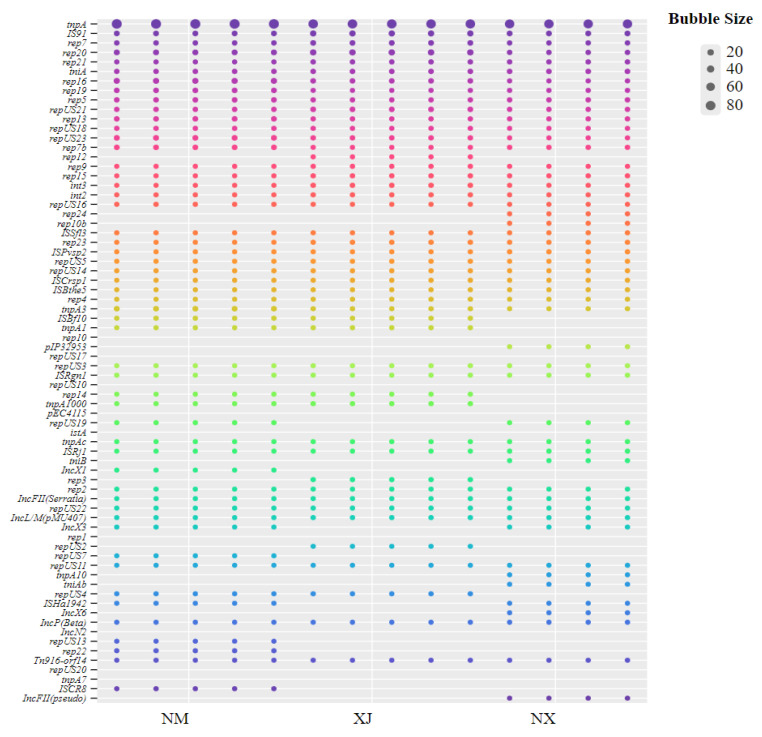
Distribution of MGEs in cattle feces samples from three regions.

Analysis of MGEs revealed a total of 90 distinct MGE genes (Figure 7), and tnpA had the highest prevalence across the 3 pastoral areas. In contrast, IncFII and *rep*US showed a geographical distribution pattern, predominantly clustering in the Ningxia and Inner Mongolia pastoral areas with relatively high antibiotic residues. Moreover, rep12 was only annotated in fecal samples from the Xinjiang area.

### 3.5. Analysis of Co-Correlation of ARGs, MGEs, and HBPs

**Figure 8:**
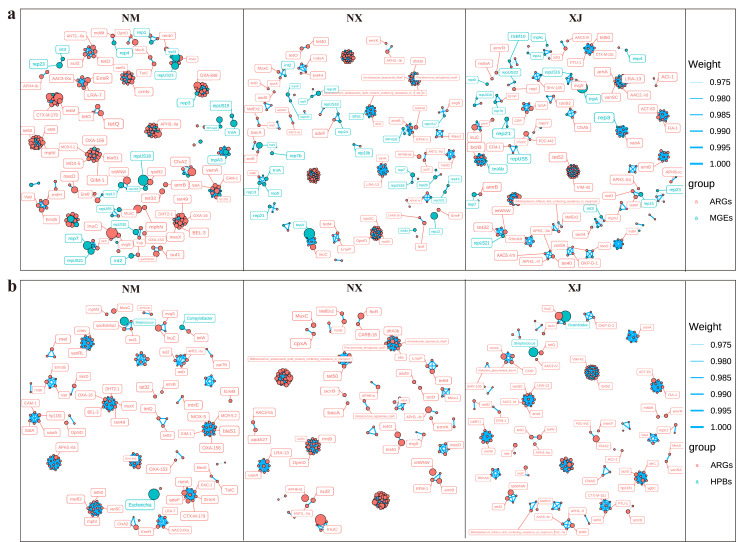
The co-occurrence network visualizes the direct relationships between genus-level hosts (**a**), HPB hosts (**b**), and ARG subtypes. Node colors represent the modules of different microbial groups. Larger size indicates a higher degree and more edge connections. The thickness of the blue line represents the absolute value (Weight) of the correlation coefficient between two modules.

Co-correlation networks are well-suited to detect general patterns in highly populated taxonomic groups. Thus, in the present study, the co-occurrence patterns between ARG subtypes and HPBs and MGEs were also investigated using the network analysis approach (Figure 8). We hypothesized that the nonrandom co-occurrence patterns between ARGs and HPBs/MGEs could indicate the possible host information of ARGs. A co-occurrence network of ARGs with MGEs or HPBs was constructed for fecal samples across all pastoral regions (Spearman’s *ρ* > 0.7 and *p* < 0.01). Results showed that Inner Mongolia (NM) samples exhibited the highest co-occurrence rate between ARGs and MGEs, whereas Xinjiang (XJ) samples had the lowest correlation rate, indicating the strongest potential transferability of ARGs in Inner Mongolia samples (Figure 8a).

As depicted in Figure 8a, *rep* plasmids formed the most edges with ARGs across all three pastoral regions (Xinjiang, Ningxia, and Inner Mongolia), with the majority of these ARGs linked to HPBs. The *tnp* transposase family also showed pronounced associations with ARGs in northwestern pastoral areas. Co-occurrence network analysis revealed that HPBs carried 14–18 ARGs in all regions, with regional variations: Inner Mongolia samples harbored 15 ARGs associated with *Campylobacter*, *Bacillus*, *Streptococcus*, and *Escherichia*, Ningxia samples contained 13 ARGs linked to *Clostridium*, *Escherichia*, *Streptococcus*, and *Klebsiella pneumoniae*, and Xinjiang (XJ) samples exhibited 9 ARGs associated with *Clostridioides*, *Escherichia*, *Streptococcus*, and *Campylobacter*.

### 3.6. Association Analysis of Multidrug-Resistant HPBs and ARGs in Feces Samples

**Figure 9:**
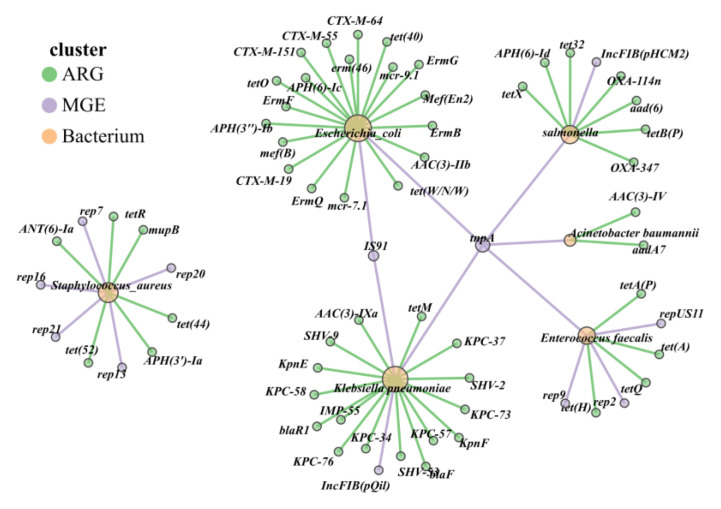
Host diversity and multi-resistance patterns of ARGs. Different colors of dots represent ARG (green), MGE (purple), and bacterium (yellow), and the different color lines show the HPBs connected with MGE (purple) or ARGs (Green).

To further elucidate the correspondence between ARGs and multidrug-resistant HPBs in the cattle gut microbiota of three pastoral provinces in northwestern China, the top five most abundant HPBs and their associated ARGs and MGEs in fecal samples were analyzed. Analysis identified 5 animal-derived bacterial species of high concern to the WHO, each carrying multiple ARGs: *Pseudomonas aeruginosa* (7 ARGs), *Escherichia coli* (19 ARGs), *Klebsiella pneumoniae* (17 ARGs), *Clostridioides difficile* (5 ARGs), and *Staphylococcus aureus* (6 ARGs; Figure 9). ARGs in *Escherichia coli* and *Klebsiella pneumoniae* primarily conferred resistance to tetracyclines, extended-spectrum β-lactams (ESBLs), carbapenems, and polymyxins, whereas the remaining three multidrug-resistant organisms (MDROs) harbored ARGs against aminoglycosides, macrolides, and other antibiotic classes.

Mobile genetic elements (MGEs) act as accelerators for the spread of ARGs. The IncFIB plasmid in the fecal samples of grazing cattle from the western pastoral areas was the main carrier for the transmission of carbapenem resistance genes. This indicates that *Klebsiella pneumoniae* had a risk of transferring resistance genes to other bacteria through conjugative plasmids, giving rise to new multidrug-resistant organisms (MDROs). TnpA and IS91 facilitate horizontal gene transfer of ARGs between *Escherichia coli* and *Klebsiella pneumoniae*, while rep-family replicons primarily mediate the dissemination of ARGs in *Clostridioides difficile* and *Staphylococcus aureus* (Figure 9).

## 4. Discussion

The use of antibiotics in animal husbandry is a significant contributor to the emergence of antibiotic resistance. A survey in Ningxia’s Guyuan dairy farm identified tetracyclines (e.g., doxycycline and oxytetracycline), quinolones, and sulfonamides as dominant fecal antibiotics in penned cattle, with tetracycline residues exceeding other antibiotics by >1000 μg/kg throughout the breeding cycle [33]. Our study found high levels of tetracycline, β-lactam, and quinolone residues in cattle intestines from three Northwestern China pastoral areas (Figure 2), reflecting frequent use of these antibiotics in local grazing regions. A report showed tetracyclines and sulfonamides as the predominant components in water and soils around the livestock breeding areas [34]. More than 70% of tetracycline compounds were excreted in their original form or feces [35]. Our study also found relatively high detection levels of tetracycline-related antibiotics other than tetracycline in the feces (Figure 2), most likely due to their metabolic characteristics, leading to large-scale excretion. Moreover, our results also showed that there were some differences in the type and concentration of antibiotic residues in the three regions (Figure 2), as the previous surveys stated [36]. We speculate that the differences in antibiotic residues in bovine samples from the northwestern region are mainly due to the incidence rate, disease type, workers’ experience, and antibiotic properties in the farm.

This research revealed that in the cattle fecal samples collected from the grazing areas in northwest China, the phyla *Bacillota*, *Pseudomonadota*, *Bacteroidota*, and *Euryarchaeota* were the dominant ones across the three regions, collectively constituting over 90% of the microbiota (Figure 3). Peng et al. [37] investigated the bacterial composition in the feces of farmed animals in the Ningxia region. The results showed that *Bacillota*, *Pseudomonadota*, *Actinobacteriota*, and *Bacteroidota* were the four dominant phyla in the feces of all animals, which is highly consistent with our survey results. A survey in northern China found that the dominant phyla of the same farm were different in the feces, wastewater, and soils, indicating differences in bacterial species abundance among different environmental samples [38]. *Bacteroides* are one indicator of changes in the gut microbiota [39,40]. The utilization of tetracycline-class drugs frequently results in an elevated abundance of *Bacteroides* [41]. In this study, the residual concentration of tetracycline-class antibiotics in the samples from the Xinjiang region was notably lower than that in other pastoral areas. Correspondingly, the abundances of numerous microorganisms, including those from the phylum *Bacteroidota*, were also comparatively low in the Xinjiang samples (Figure 3). This finding suggests that tetracycline-class antibiotics exerted a substantial influence on the composition of the microbiota in the grazing cattle.

Recent studies show that livestock grazing not only introduces ARM directly into the soil through manure but also leads to the interaction of ARM in livestock, livestock excreta, soil, and plant platforms [20,21], which poses a potential risk of ARM spreading. Also, studies have found that livestock manure from grazing activities leads to changes in river water quality in pastoral areas, reducing river biological integrity and downstream water quality [42]; however, due to the infiltration of surface water and soil materials, groundwater is easily contaminated by antimicrobial agents and antibiotic resistance bacteria (ARBs) in the surrounding environment [25]. In addition to the widespread negative environmental impacts, water contaminated with livestock manure can also lead to harmful health effects and may spread diseases in pastoral areas, posing a threat to human health [42]. ARG can be transformed from animal bacteria into human pathogens through horizontal gene transfer (HGT; such as binding, transformation, and transduction) mediated by mobile genetic elements (MGEs). MGEs, such as plasmids, transposons, and integrons, facilitate the horizontal gene transfer (HGT) of ARGs among different bacteria [43]. Once an ARG is introduced into opportunistic human bacterial pathogens, it will lead to the occurrence of human diseases and the failure of antibiotic treatment [44,45]. Our study found that the most abundant MGEs in the fecal samples of grazing cattle from Inner Mongolia, Xinjiang, and Ningxia were plasmids, transposases, and integrases. As many as 90 types of MGEs were detected, indicating a high risk of horizontal transfer of ARGs.

The atmosphere and water have been recognized as an important hotspot for ARGs and ARBs [46]. Liu et al. [47] found that 88.9% of *E. coli* in surveyed pig farm wastewater carried tetracycline-resistant genes, directly linked to widespread tetracycline use, with contaminated feces exacerbating environmental antibiotic resistance spread. Moreover, various typical anthropogenic repositories of airborne ARGs have been recognized, including hospitals, livestock farms, landfills, and wastewater treatment plants (WWTPs) [48,49,50,51]. Agarwal et al. [52] analyzed air samples from a dairy farm and detected ARGs such as *tet*W, *aad*A1, and *sul*2, and MGEs, including *intl*1, *tnp*A, and *IS*26, were highly consistent in the cattle manure samples, indicating that cattle feces are a major source of air pollution. A recent comparative study also revealed that livestock air is a significant ARG exposure pathway for farm workers and surrounding residents, and farm air ARGs are highly transferable to human pathogens [53]. Pathogens carrying ARGs pose a greater threat to human health [54]. The American CDC monitored the exposure to water from 2013 to 2014, finding at least 289 cases of illness, 108 hospitalizations, and 17 deaths, proving a dramatic public health risk from environmental exposure to ARBs [54]. The pastoral areas in northwest China cover a vast expanse of land, which greatly increases the uncertainty of the transmission of ARGs and HPBs through animal feces via the water, air, and soil. The risk of ARGs in grazing cattle being transmitted to humans through aerosols and the food chain deserves our serious attention.

An analysis of ARGs and MGEs in hospital wastewater in the Shanghai region showed that the dominant bacterial species in the wastewater were *Escherichia coli*, *Aeromonas hydrophila*, *Klebsiella pneumoniae*, and *Aeromonas* spp., all of which are zoonotic pathogens. Moreover, the samples were rich in MGEs, such as plasmids, transposons (*tnp*A), integrons (*intI*1), and insertion sequences (IS91) [55]. This indicates the risk of ARGs in these pathogens being transmitted utilizing the above-mentioned MGEs, presenting challenges for clinical treatment. Our study demonstrated that IncFIB serves as a transmission vector for the resistance genes of *Klebsiella pneumoniae*. Additionally, *tnp*A and *IS*91 facilitate the horizontal transfer of resistance genes between *Escherichia coli* and *Klebsiella pneumoniae*. The *rep* series replicons primarily mediate the dissemination of ARGs in *Clostridioides difficile* and *Staphylococcus aureus*. These findings indicate that MGEs such as *tnp*A, IS91, and *rep* plasmid are involved in the transfer of ARGs among human bacterial pathogens, and the associated clinical risks cannot be overlooked. Herein, we found that *Escherichia coli* and *Klebsiella pneumoniae* are two important zoonotic pathogens, carrying a variety of crucial ARGs, such as carbapenem-class ARGs and β-lactamase-class ARGs (Figure 9). There have been frequent reports of them acting as human pathogens. Among the carbapenem-resistant *Enterobacteriaceae* bacteria isolated from clinical samples in hospitals across 24 provinces, researchers found that *Klebsiella pneumoniae* had the highest carrying rate of *bla*_KPC-2_, while in *Escherichia coli*, the most commonly found was *bla*_NDM_ [56].

Therefore, it is of great significance to adopt metagenomic analysis methods and establish an early warning system for the above-mentioned pathogens in grazing animals and their surrounding environments.

## 5. Conclusions

The effects of antibiotic residues, MGEs, and bacterial communities on ARGs in cattle feces from the three northwest Chinese grazing provinces (Xinjiang, Ningxia, and Inner Mongolia) were studied using HPLC-MS and metagenomic sequencing. The results showed a significant negative correlation between antibiotic residues and microbial abundance and a significant positive correlation with the prevalence of ARGs. Notably, we identified carbapenemase-resistant genes (KPC/VIM) at alarmingly high levels, and a disease-associated pathogen—*Klebsiella pneumoniae*—with a high possibility of carrying *bla*_CTX-M-55_ included in IncFII plasmids harboring transposons (*tnp*A) and insertion sequences (*IS*91), indicating a horizontal gene transfer risk of carbapenemase-resistant genes over grazing areas. However, there were differences in ARG subtypes. This study explored the association between important drug-resistant bacteria with high clinical attention and drug-resistant genes in the cattle breeding process with grazed modes, which can guide the tracing of drug-resistant genes, rational drug use, and prevention and control of bacterial diseases.

## 6. Limitations

The data represent a single sampling season (August to October 2023) and focus only on the cattle feces. Consequently, our findings may not capture potential seasonal variations, long-term trends, or the full impact of episodic events on contaminant levels or environmental dynamics in soil, water, and air. In the future, monitoring should design a more comprehensive sampling strategy to learn the influences of antibiotic residues, MGEs, and bacterial communities on ARGs in cattle grazing areas in the three northwest Chinese provinces.

## Data Availability

All sequences have been submitted to the National Center for Biotechnology Information (NCBI) database under the BioProject accession number PRJNA1260217.

## Supplementary Materials

The following supporting information can be downloaded at: https://www.mdpi.com/article/10.3390/microorganisms13071658/s1, Figure S1: The environmental metadata from August to October of 2023; Table S1: Matrix effects (ME) of target components in fecal matrix.; Table S2: Limits of detection (LOD) and quantification (LOQ) for each antibiotic.; Table S3: The relative abundance of bacterial phyla in the cattle fecal samples.; Table S4: The relative abundance of genera in the cattle fecal samples.; Table S5: The relative abundance of ARGs in the cattle fecal samples.; Table S6: The relative abundance of MGEs in the cattle fecal samples.
