# Supplementary material for: Effects of Antibiotic Residues on Fecal Microbiota Composition and Antimicrobial Resistance Gene Profiles in Cattle from Northwestern China
Source: Microorganisms. 2025 Jul 14;13(7):1658. doi: 10.3390/microorganisms13071658 (PMC12298114; doi:10.3390/microorganisms13071658)
Supplement: Supplementary file 1 [file microorganisms-13-01658-s001.zip › microorganisms-3695428 SUPPLEMENTAL MATERIALS-proof.pdf]

SUPPLEMENTAL FIGURES

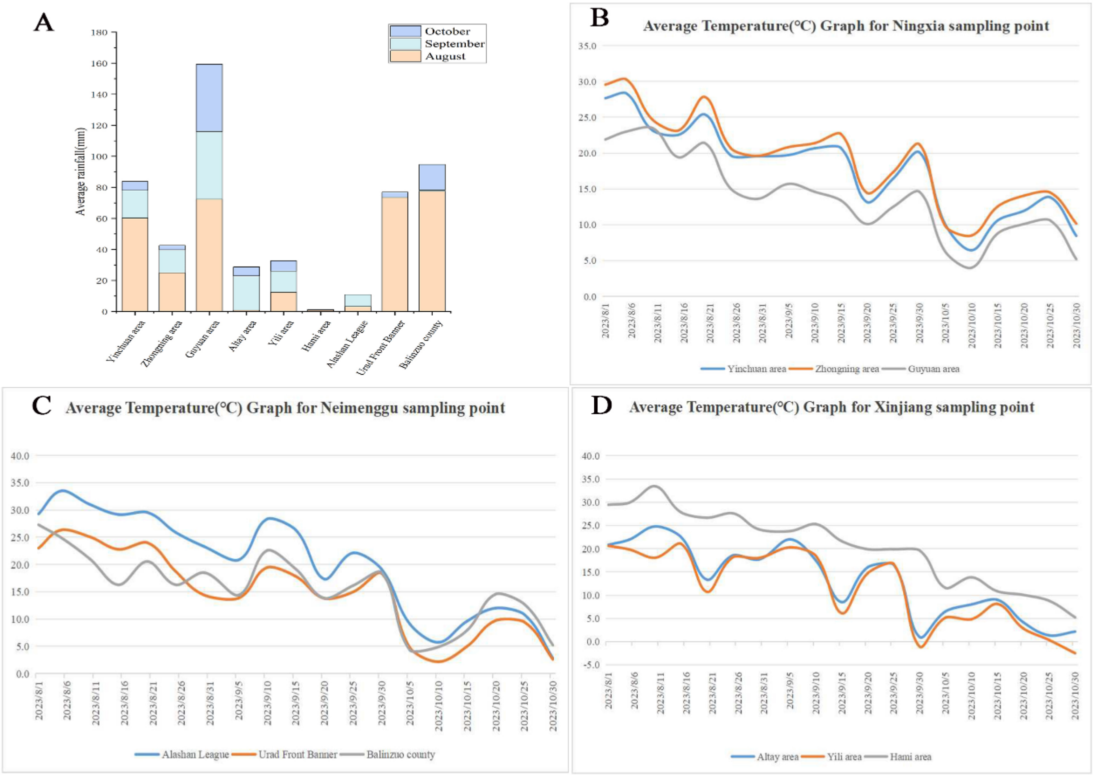

Figure S1. **The environmental metadata from August to October of 2023.** A) The average rainfall of the cities involved in the sampling. The average temperature (°C) graph for the sampling point of Ningxia province(B), Inner Mongolia(C), and Xinjiang(D).

SUPPLEMENTAL TABLES

Table S1. Matrix effects (ME) of target components in fecal matrix.

Compound	Linear range(ng/mL)	Linear Equation	r	ME(%)	Recoveries(RSDs)%		
					4 μg/kg	20 μg/kg	32 μg/kg
Oxytetracycline（OXY）	2.0～100.0	*Y*=589*X*-781	0.993	6.2	77.5(5.6)	71.1 (9.8)	72.9(9.9)
Tetracycline (TE)	2.0～100.0	*Y*=255*X*-974	0.996	3.6	68.0(7.2)	61.1 (11.2)	62.0(10.6)
Chlortetracycline (CTE)	2.0～100.0	*Y*=1099*X*-569	0.999	5.8	75.6(6.4)	77.1 (6.8)	75.1(8.6)
Doxycycline（DOX）	2.0～100.0	*Y*=8777*X*-11126	0.998	3.8	77.4(5.9)	81.2(5.2)	83.2(6.0)
Ampicillin(AMP)	2.0～100.0	*Y*=7896*X*-12313	0.999	20.0	85.8(5.3)	86.0 (7.3)	88.5(7.5)
Cefazolin(CZO)	2.0～100.0	*Y*=4555*X*-5786	0.993	12.5	71.9(7.2)	72.6 (6.3)	74.7(6.1)
Penicillin G (PEN)	2.0～100.0	*Y*=5263*X*-4720	0.999	6.2	75.9(6.5)	78.9(6.3)	77.8(6.5)
Cefuroxime（TIO）	2.0～100.0	*Y*=4878*X*-1359	0.992	9.7	81.8(4.2)	83.9 (3.7)	87.5(6.0)
Ciprofloxacin（CIP）	2.0～100.0	*Y*=1988*X*-5541	0.991	5.0	82.6(5.7)	81.8 (4.6)	80.5(4.2)
Ofloxacin（OFX）	2.0～100.0	*Y*=7879*X*-5231	0.998	9.2	78.1(4.4)	80.8 (5.4)	79.9(5.2)
Enrofloxacin(EIN)	2.0～100.0	*Y*=1540*X*-2101	0.998	6.1	85.9(5.5)	86.7(4.2)	88.0(5.5)
Trimethoprim（TMP）	1.0～100.0	*Y*=40895*X*-56700	0.999	11.1	79.4(3.7)	81.8 (3.6)	82.5(5.2)
Sulfadiazine（SDI）	2.0～100.0	*Y*=5158*X*-4501	0.999	2.6	74.3(8.7)	71.6 (5.5)	73.6(6.1)
Colistin（COL）	2.0～100.0	*Y*=10882*X*-34031	0.998	5.2	78.1(9.8)	71.8 (8.4)	80.6(7.5)
Gentamicin(GEN)	2.0～100.0	*Y*=12633*X*-9125	0.998	2.9	82.4(5.7)	87.9 (7.0)	85.4(6.9)
Metronidazole (MTR)	1.0～100.0	*Y*=12633*X*-9125	0.997	2.9	82.4(5.7)	87.9 (7.0)	85.4(6.9)
Lincomycin (LIN)	2.0～100.0	*Y*=13606*X*-82631	0.993	2.6	75.2(7.5)	69.3 (9.3)	70.3(8.5)
Griseofulvin (GRI)	2.0～100.0	*Y*=7099*X*-13890	0.997	4.9	72.1(5.5)	68.6 (4.2)	75.3(4.6)

Table S2. Limits of detection (LOD) and quantification (LOQ) for each antibiotic.

Compound	LOD(μg/kg)	LOQ(μg/kg)
Oxytetracycline（OXY）	0.5	2
Tetracycline (TE)	0.5	2
Chlortetracycline (CTE)	0.5	2
Doxycycline（DOX）	0.5	2
Ampicillin(AMP)	0.5	1
Cefazolin(CZO)	0.5	2
Penicillin G (PEN)	0.5	1
Cefuroxime（TIO）	0.5	2
Ciprofloxacin（CIP）	0.5	2
Ofloxacin（OFX）	0.5	2
Enrofloxacin(EIN)	0.5	2
Trimethoprim（TMP）	0.1	0.5
Sulfadiazine（SDI）	0.5	2
Colistin（COL）	0.5	2
Gentamicin(GEN)	0.5	1
Metronidazole (MTR)	1	4
Lincomycin (LIN)	0.5	2
Griseofulvin (GRI)	0.5	1

Table S3. The relative abundance of bacterial phyla in the cattle fecal samples.

This table is provided in a separate Excel file (TableS3-S6.xlsx). The table contains the relative abundance of bacterial phyla (Fig. 3C).

Table S4. The relative abundance of genera in the cattle fecal samples.

This table is provided in a separate Excel file (TableS3-S6.xlsx). The table contains the relative abundance of bacterial phyla (Fig. 3A and Fig. 3D).

Table S5. The relative abundance of ARGs in the cattle fecal samples.

This table is provided in a separate Excel file (TableS3-S6.xlsx). The table contains the relative abundance of ARGs of each sample (Fig. 5A).

Table S6. The relative abundance of MGEs in the cattle fecal samples.

This table is provided in a separate Excel file (TableS3-S6.xlsx). The table contains the relative abundance of MGEs of each sample (Fig. 5D).
